# Label free technologies 3: infrared imaging applied to paraffinized tissue microarrays for colon cancer diagnosis

**DOI:** 10.1186/1746-1596-8-S1-S34

**Published:** 2013-09-30

**Authors:** Ganesh D Sockalingum, Jayakrupakar Nallala, Marie-Danièle Diebold, Cyril Gobinet, Olivier Piot, Valérie Untereiner, Michel Manfait

**Affiliations:** 1 MEDyC, FRE CNRS/URCA 3481, France; 2 CHU Reims, France

## Introduction

Colorectal cancers are the third most common type of cancers globally, affecting both sexes [1]. As of now, histopathology is the gold standard method for colon cancer diagnosis. Newer technologies are the important need of the hour to complement the existing approaches, for better understanding the onset and progression of the disease. Infrared (IR) imaging could be a potential candidate method because of its capability to probe non-destructively and in a label-free manner the intrinsic chemical bonds present in the tissue, thus giving a “spectral fingerprint” of its composition and structures. To this end, we have developed IR spectral histopathology with the aims to: (a) examine, the molecular changes between normal and tumoral colon tissues, (b) exploit its potentials to identify new diagnostic markers to complement conventional histopathology, and (c) develop an algorithm as an automatic diagnostic tool for tumor prediction, directly on paraffinized samples, without chemical de-waxing, staining or any further preparation.

## Methods

Nine normal and 25 tumoral colon tissue sections (3mm diameter x 10 µm thick) embedded in the form of paraffinized tissue microarray, stabilized in an agarose matrix were analyzed directly by IR imaging. To avoid chemical deparaffinization, a modified Extended Multiplicative Signal Correction (EMSC) method[2] was used to digitally neutralize the spectral interferences of paraffin and agarose, and to preserve only the biological information from the tissue. Corrected spectra were classified using k-means to construct color-coded images using the HPS stained sections as reference. Linear Discriminant Analysis (LDA) was then used to construct a prediction model for identification of blind samples.

## Results

EMSC permitted mathematical correction of the spectral interferences originating from paraffin and agarose. K-means classification allowed to identify and to distinguish important histological features of the colonic tissues such as crypts, lamina propria, tumor, etc. When compared to HPS stained images, after whole slide image analyzed with crop and score Calopix module from TRIBVN or through pathologist control, color-coded spectral images not only reveal features representative of the biochemical make up of the tissues, but also highlight additional features like intra-tumoral heterogeneity and tumor-associated stroma, which are difficult to discern by conventional histopathology. The LDA prediction model was promising since an average sensitivity of 91 % was achieved in the identification and prediction of tumoral tissues.

## Discussion

IR imaging allowed differentiating and detecting normal and tumoral colon tissue features based on their intrinsic biochemical information. This chemical-free approach on paraffinized tissue biopsies combined with multivariate statistical image analysis opens a new avenue for numerical spectral histopathology and appears as a promising tool for colon cancer diagnosis. Further work to improve the model and to predict tumors in blind samples is ongoing.
